# Efficacy and safety of voriconazole and caspofungin for the treatment of invasive pulmonary aspergillosis in critically ill patients in China

**DOI:** 10.3389/fcimb.2025.1584950

**Published:** 2025-05-21

**Authors:** Zhuo Li, Yuan Zong, Dayu Chen, Juan Ma, Peng Zhang, Lu Cao

**Affiliations:** ^1^ Department of Pharmacy, Shaanxi Provincial People’s Hospital, Xi’an, Shaanxi, China; ^2^ Department of Intensive Care Unit, Shaanxi Provincial People’s Hospital, Xi’an, Shaanxi, China; ^3^ Department of Pharmacy, Nanjing Drum Tower Hospital, Nanjing, Jiangsu, China; ^4^ Department of Clinical Laboratory, Shaanxi Provincial People’s Hospital, Xi’an, Shaanxi, China

**Keywords:** invasive pulmonary aspergillosis, safety, efficacy, voriconazole, caspofungin

## Abstract

**Objective:**

To compare and analyze the efficacy and safety of different antifungal drug treatment regimens for patients with invasive pulmonary aspergillosis (IPA) in the intensive care unit (ICU).

**Methods:**

We retrospectively collected the clinical data of patients with IPA in the ICU in two grade-A tertiary hospitals from January 2019 to January 2024 using the HIS system and compared the clinical efficacy, incidence of adverse drug reactions (ADRs), and all-cause mortality at discharge among different antifungal treatments, such as voriconazole alone, caspofungin alone, and a combination of voriconazole plus caspofungin.

**Results:**

A total of 151 patients were enrolled, including 129 in the monotherapy group (with 92 in the voriconazole subgroup and 37 in the caspofungin subgroup) and 22 in the voriconazole plus caspofungin combination group. *Aspergillus fumigatus* was the most common pathogenic fungus in patients with IPA, followed by *Aspergillus flavus*. In terms of clinical efficacy, monotherapy and combination therapy were equally effective (*P*=0.618), and the efficacy of voriconazole or caspofungin alone and that of voriconazole combined with caspofungin in the treatment of IPA was equivalent (*P*=0.630). In terms of safety, the total incidence of ADRs in the combination therapy group was greater than that in the monotherapy group, but the difference was not statistically significant (*P*=0.109). The two groups were also equally safe in terms of causing renal dysfunction, liver dysfunction, visual abnormalities, and hypokalemia. However, compared with the monotherapy group, the combination therapy group exhibited a significantly greater incidence of pancytopenia (*P*=0.013, *P*=0.004). The all-cause mortality in the combination therapy group was significantly greater than that in the monotherapy group (*P*=0.027, *P*=0.009).

**Conclusions:**

Voriconazole is still the preferred treatment for critically ill patients with IPA, and caspofungin has good clinical efficacy and safety and can effectively replace voriconazole for these patients. However, the combination treatment with voriconazole and caspofungin did not improve the all-cause mortality rate of critically ill patients with IPA, is associated with increased total ADR and pancytopenia incidence and is not recommended as an initial treatment plan.

## 1 Introduction

Invasive fungal disease (IFD) refers to a severe fungal infection in which fungi invade the human body; proliferate in tissues, organs, or blood; and cause severe inflammation or tissue damage. These are life-threatening fungal infections with a high mortality rate. In recent years, the incidence of IFD has steadily increased due to the widespread use of glucocorticoids, broad-spectrum antibiotics, and immunosuppressive agents in clinical practice. Patients in the ICU are more susceptible to IFD due to the impairment or collapse of their physiological defense mechanisms, which can exacerbate their original diseases. Research shows that the overall case fatality rate of these patients exceeds 80% (Chinese Society of Critical Care Medicine, 2007).

*Aspergillus* is a fungal genus that is widely distributed in the natural environment, and these fungi have strong adaptability and transmissibility. These microorganisms infiltrate the human body through the respiratory tract, causing aspergillosis (Guo et al., 2023). Among these species, *Aspergillus fumigatus* is the most common pathogen, accounting for approximately 70~80% of the reported cases (Walsh et al., 2008). Other non-*A. fumigatus* species include *A. flavus, A. niger, A. nidulans, A. terreus*, and *A. versicolor (* Cadena et al., 2016; Yii et al., 2017). Despite significant progress, aspergillosis remains a serious health problem, with its epidemiology constantly evolving and the population of high-risk patients expanding continuously (Herrera et al., 2025). Invasive pulmonary aspergillosis (IPA) is the most severe form of *Aspergillus* invasion and is also the most common type of invasive fungal infection in ICU patients (Liu et al., 2017; Herrera et al., 2025). IPA carries a significant risk of death and imposes a substantial burden on affected individuals (Quindós, 2014). Improving the clinical prognosis of IPA depends on two key factors: the accuracy of diagnosis and the precision of antifungal treatment. However, unfortunately, owing to the nonspecificity of symptoms, the diagnosis of IPA is extremely challenging (Tunnicliffe et al., 2013). Moreover, for some diagnostic techniques, such as sputum culture, considerable effort is needed to obtain a result (Donnelly et al., 2020). Therefore, clinicians often provide empirical treatment for patients suspected of having IPA, which severely limits the effectiveness of clinical treatment.

The guidelines for the treatment of fungal infections in adult respiratory and ICU Patients published by the American Thoracic Society (ATS) in 2011 recommended prompt and aggressive antifungal therapy upon clinical suspicion or diagnosis of IPA (Limper et al., 2011). The 2016 version of the clinical practice guidelines on *Aspergillus* infections by the Infectious Diseases Society of America (IDSA) recommended voriconazole as the first-line treatment for IPA, with alternative and salvage therapies including amphotericin B, itraconazole, or caspofungin. Combination therapy with voriconazole and an echinocandin is generally reserved for salvage treatment when the initial therapy is ineffective (Patterson et al., 2016).

The current recommendations for IPA treatment are based primarily on evidence from patients with hematological disorders or malignancies, leaving a gap in recommendations based on evidence from other IPA populations, such as patients in the ICU (Zheng, 2018; Yang et al., 2021). Given that the treatment of IPA is mostly empirical in ICUs, with relatively low cure rates and high mortality rates, clinicians are increasingly inclined to use combination therapy (Yang et al., 2021). Studies have reported that up to 30% of critically ill IPA patients require salvage treatment (Wang and Xu, 2019; Han, 2020; Yang et al., 2021). However, there is limited clinical experience regarding the efficacy and safety of alternative regimens and combination therapy for IPA. Therefore, this study aimed to include patients who were clearly diagnosed with IPA on the basis of the results of fungal culture. First, in this study, we aimed to understand the epidemiological characteristics of *Aspergillus* in the local area. Second, we compared the efficacy and safety of voriconazole monotherapy, caspofungin monotherapy, and combination therapy with voriconazole plus caspofungin treatment in critically ill IPA patients to provide a reference for clinical practice.

## 2 Materials and methods

### 2.1 Study design and eligibility criteria

This retrospective multicenter real-world study included patients diagnosed with IPA based on the European Organization for Research and Treatment of Cancer and Mycoses Study Group Education and Research Consortium (EORTC/MSGERC) consensus definitions of IFD (Donnelly et al., 2020; Alexander et al., 2021); the patients were treated at two Grade-A tertiary hospitals between January 2019 and January 2024. The inclusion criteria for the patients were as follows: (1) aged ≥ 18 years; (2) admitted to ICUs; (3) positive culture of *Aspergillus* species (Cadena et al., 2016) (e.g., *A. fumigatus*, *A. flavus, A. niger, A. terreus, etc.*) from sputum or bronchoalveolar lavage fluid; (3) initial antifungal treatment via monotherapy, including voriconazole alone and caspofungin alone, or combination therapy with voriconazole plus caspofungin, at doses consistent with guidelines and drug labels; and (4) duration of antifungal therapy ≥ 3 days (Cao et al., 2023). The exclusion criteria for patients were as follows (Li et al., 2024; Yuan et al., 2025): (1) patients with documented allergies to voriconazole or caspofungin; (2) patients with *Aspergillus* genus that had not been identified to the species level; (3) patients who were pregnant or breastfeeding; (4) patients with other severe infectious diseases; (5) patients who were participating in other clinical studies simultaneously; and (6) patients with incomplete medical records or missing clinical laboratory data.

The identification of *Aspergillus* species was carried out using matrix-assisted laser desorption/ionization time-of-flight mass spectrometry (MALDI-TOF MS) (Zhang et al., 2023), and the specific operation steps are described below. (1) The method for extraction with formic acid and acetonitrile was as follows: Colonies were cultured on potato dextrose agar (PDA) for 3 days were scraped with an inoculation loop. The mycelia at the edge were picked and added to a mixture of 300 μL of pure water and 900 μL of absolute ethanol. The mixture was shaken and then centrifuged at 13,000 r/min for 2 minutes. The supernatant was discarded, and the residue was dried. Then, 50 μL of 70% formic acid was added, and the mixture was shaken for 20 seconds at 4,000 r/min to break the cell wall. This process was repeated twice, after which the mixture was allowed to stand for 5 minutes. Then, 50 μL of acetonitrile was added, and the mixture was left to stand for 5 minutes. The mixture was centrifuged at 13,000 r/min for 2 minutes, and the resulting supernatant was used as the fungal protein template. (2) The modified method of extraction with zirconium bead grinding, formic acid and acetonitrile was as follows: young mycelia cultured on PDA for 2 days were scraped with an inoculation loop, added to 1 mL of potato broth, and shaken horizontally on a shaker at 28°C for 24 hours to enrich the fungi, which formed dense colonies visible to the naked eye. Two zirconium beads were added, and the remaining operations were performed as described for the extraction method with formic acid and acetonitrile. (3) Mass spectrometry was performed as follows: one microliter of fungal protein was used to spot the target plate. After air-drying, the sample was covered with 1 μL of matrix. After air-drying again, mass spectrometry identification was performed. (4) Identification scoring was performed as follows: the target plate was spotted twice for each fungal strain for identification. The names of the fungi and the identification scores were recorded when the results were consistent. The identification criteria were as follows: the score for identification at the species level was ≥ 2.0 points; if the score was lower than 2.0 points but the top 5 results from all identifications were for the same fungal species and the score was between 1.7 and 2.0 points, identification was considered successful at the genus level; if the score was lower than 1.7 points or if the identification results included multiple fungal species, identification was considered unsuccessful.

### 2.2 Variables and definitions

We collected detailed information from medical records, including information on demographics (e.g., age, sex, and underlying conditions), clinical characteristics (Acute Physiology and Chronic Health Evaluation II (APACHE II) scores, symptoms, imaging results, blood gas parameters, oxygenation indices), biochemical examinations (liver and kidney function (e.g., serum creatinine, alanine aminotransferase (ALT), aspartate aminotransferase (AST), alkaline phosphatase (ALP), total bilirubin (TB) and conjugated bilirubin (CB) levels), routine blood tests (white blood cell (WBC), neutrophil, hemoglobin (HB) and platelets (PLT) levels), fungal culture results (e.g., fungal detection, sampling and clearance), antifungal treatment regimens and durations, and any recorded adverse drug reactions (ADRs).

Patients were grouped into a voriconazole monotherapy group, a caspofungin monotherapy group, and a combination therapy group treated with voriconazole plus caspofungin. Only the initial therapy regimen was considered for the analysis of clinical efficacy and safety if treatment regimens were subsequently changed during therapy.

### 2.3 Clinical outcomes and criteria

Clinical outcomes at discharge, including clinical efficacy, all-cause mortality and ADRs, were also assessed.

Safety was evaluated on the basis of ADRs, e.g., liver function, kidney function, routine blood test results, and other ADRs recorded before and after antifungal therapy. Renal toxicity was defined as an increase in serum creatinine level of at least 0.55 mg/dL or a rise of ≥50% from baseline (Cao et al., 2022). Liver damage was diagnosed when AST, ALP, and TB levels were simultaneously elevated, with at least one exceeding twice the upper limit of normal (ULN), or when the ALT or CB level exceeded twice the ULN after ruling out viral hepatitis and other causes of liver injury (Yu et al., 2017). Hematological toxicity was diagnosed as reductions in WBC, HB, and PLT counts to below normal reference ranges after antifungal therapy (Wang et al., 2021). Other ADRs, such as nausea, vomiting, rash, diarrhea, visual disturbances, and hypokalemia, were identified on the basis of drug label descriptions.

According to the published literature (Yang et al., 2021), clinical efficacy was classified into three levels: the patient was considered cured if symptoms, laboratory tests, and imaging findings were normal and all pathogens had been cleared. The treatment was considered markedly effective if significant improvement in symptoms and laboratory tests was observed, with ≥50% improvement on imaging, but with some persisting abnormalities. The treatment was considered ineffective if no significant improvement or worsening of clinical condition or imaging findings, or even death, was observed. The total efficacy of antifungal treatment = (number of cured patients + number of patients with marked effect)/total number of patients × 100%.

### 2.4 Statistical analysis

Statistical analysis was performed using SPSS 23.0 software. Categorical variables are expressed as frequencies and percentages, with group comparisons via the χ^2^ test or Fisher’s exact test. Continuous variables conforming to a normal distribution are expressed as x ± s, with group comparisons performed via one-way ANOVA. The ordinal variables were compared using the *Mann–Whitney U test*. The Logistic regression analysis method was used to evaluate the risk factors associated with all-cause mortality. First, the univariate Logistic regression analysis method was employed. By calculating the P - value, odds ratios (ORs), and 95% confidence intervals (95% CI), the correlation between confounding factors and all-cause mortality was initially explored, especially the impact of antifungal treatment regimens on it. Second, the multivariate Logistic regression analysis method was used. The antifungal treatment regimens and the confounding factors with P < 0.05 in the univariate analysis results were included in the multivariate analysis. A stepwise multiple regression analysis model was adopted for analysis to identify the independent predictors associated with all-cause mortality, especially the impact of antifungal treatment regimens on it. The strength of the correlation between independent risk factors and all - cause mortality was evaluated by calculating the adjusted odds ratios (adjusted ORs) and 95% confidence intervals (95% CI). A p value of <0.05 was considered to indicate statistical significance.

## 3 Results

### 3.1 Clinical characteristics

A total of 151 patients were included in this study. The patients were divided into a monotherapy group, which included 129 patients (85.43%), consisting of a voriconazole monotherapy subgroup and a caspofungin monotherapy subgroup, and a combination therapy group, which included 22 patients (14.57%). The voriconazole monotherapy subgroup included 92 patients (64 males, 28 females) with a mean age of 68.48 ± 13.85 years, an average treatment duration of 7.48 ± 5.31 days, and a mean APACHE II score of 15.64 ± 7.24. The most common underlying conditions were type 2 diabetes and chronic obstructive pulmonary disease (COPD). The caspofungin monotherapy subgroup included 37 patients (22 males, 15 females) with a mean age of 67.89 ± 14.14 years, an average treatment duration of 8.92 ± 6.26 days, and a mean APACHE II score of 16.24 ± 5.71. COPD was the most common underlying condition, followed by hypertension and type 2 diabetes. The combination therapy group included 22 patients (13 males, 9 females) with a mean age of 68.23 ± 11.22 years, an average treatment duration of 6.73 ± 3.03 days, and a mean APACHE II score of 18.21 ± 9.46. The common underlying conditions were COPD, renal failure, and type 2 diabetes. Approximately 50% of the patients in each of the three groups had a history of steroid use. There were no statistically significant differences (P>0.05) observed among the three groups in terms of age (*P*=0.975), sex (*P*=0.434), BMI (*P*=0.904), history of steroid use (*P*=0.722), treatment duration (*P*=0.244), underlying conditions, APACHE II score (*P*=0.073), etc. (see **Table 1** for details).

**Table 1:** Comparison of clinical characteristics among antifungal treatment regimens.

Clinical characteristics	Voriconazole monotherapy group (n=92)	Caspofungin monotherapy group (n=37)	Combination therapy group (n=22)	*F/χ^2^*	*P-*value
Age (year, $\bar{\text{x}}$ ± s)	68.48 ± 13.85	67.89 ± 14.14	68.23 ± 11.22	0.025	0.975
Female (case, %)	28 (30.43)	15 (40.54)	9 (40.91)	1.671	0.434
BMI (kg/m^2^, $\bar{\text{x}}$ ± s)	20.58 ± 7.61	21.23 ± 4.35	20.89 ± 8.77	0.987	0.904
History of steroid use (case, %)	50 (54.34)	18 (48.65)	13 (59.09)	0.652	0.722
White blood cell count (10^9^/L, $\bar{\text{x}}$ ± s)	13.45 ± 7.32	14.06 ± 5.74	13.18 ± 6.23	0.765	0.579
Neutrophil count (10^9^/L, $\bar{\text{x}}$ ± s)	9.87 ± 4.86	10.23 ± 5.78	9.98 ± 3.47	0.842	0.863
Procalcitonin (ng/mL, $\bar{\text{x}}$ ± s)	2.42 ± 1.86	3.14 ± 2.19	3.45 ± 1.73	0.799	0.814
Platelet count (10^9^/L, $\bar{\text{x}}$ ± s)	225.19 ± 123.14	203.78 ± 105.75	210.86 ± 117.52	0.652	0.248
Underlying conditions (case, %)					
Hypertension	35 (38.04)	14 (37.84)	6 (27.27)	0.932	0.628
Coronary heart disease	30 (32.61)	12 (32.43)	7 (31.82)	0.005	0.997
Type 2 diabetes	47 (51.09)	16 (43.24)	10 (45.45)	0.736	0.692
Liver failure	8 (8.70)	6 (16.22)	3 (13.64)	1.640	0.441
Renal failure	3 (3.26)	5 (13.51)	3 (13.64)	5.645	0.059
COPD	47 (51.09)	17 (45.95)	10 (45.45)	0.409	0.815
Bronchiectasis	9 (9.78)	5 (13.51)	4 (18.18)	1.311	0.519
Charlson comorbidity index ( $\bar{\text{x}}$ ± s)	4.52 ± 2.31	4.6 5± 2.27	4.94 ± 2.16	1.135	0.249
Treatment duration (day, $\bar{\text{x}}$ ± s)	7.48 ± 5.31	8.92 ± 6.26	6.73 ± 3.03	1.424	0.244
APACHE II (score, $\bar{\text{x}}$ ± s)	15.64 ± 7.27	16.24 ± 5.71	18.21 ± 9.46	3.118	0.073

### 3.2 Fungal culture results

The MALDI-TOF MS identification results of fungal cultures of the included patients were all ≥ 2.0 points. Fungal culture results revealed that *A. fumigatus* was the most common pathogen in IPA patients, with 106 patients (70.20%) testing positive. *A. flavus* ranked second, with 27 patients (17.88%), followed by *A. niger* with 7 patients (6.64%) and *A. terreus* with 6 patients (3.97%), and *A. nidulans* was the least common, with 5 patients (3.31%) (see **Table 2** for details).

**Table 2:** Comparison of fungal culture results among antifungal treatment regimens.

Strain/Cases (%)	Voriconazole monotherapy group (n=92)	Caspofungin monotherapy group (n=37)	Combination therapy group (n=22)	Total
*Aspergillus fumigatus*	71 (77.17)	19 (51.35)	16 (72.73)	106 (70.20)
*Aspergillus flavus*	12 (13.04)	12 (32.43)	3 (13.64)	27 (17.88)
*Aspergillus niger*	4 (4.35)	3 (8.11)	0 (0.00)	7 (4.64)
*Aspergillus terreus*	3 (3.26)	2 (5.41)	1 (4.54)	6 (3.97)
*Aspergillus nidulans*	2 (2.18)	1 (2.70)	2 (9.09)	5 (3.31)

### 3.3 Clinical efficacy

As shown in **Table 3**, among patients treated with monotherapy, clinical effectiveness was achieved in 50 patients, resulting in an overall effectiveness rate of 38.76%, which was slightly higher than the 36.36% observed in the combination therapy group. However, this difference was not statistically significant (*P*=0.618). The clinical effectiveness rate for voriconazole monotherapy was 41.30%, which was slightly higher than the 32.43% observed for caspofungin monotherapy and the 36.36% observed for the combination therapy. However, the differences among the three groups were not statistically significant (*P*=0.630). These results suggested that voriconazole or caspofungin monotherapy was as effective as the combination therapy for IPA treatment.

**Table 3:** Comparison of clinical efficacy among antifungal treatment regimens.

Clinical efficacy/cases (%)	Monotherapy group (n=129)		Combination therapy (n=22)	*P* value (monotherapy vs. combination therapy)	*P* value (comparison among three regimens)
	Voriconazole monotherapy group (n=92)	Caspofungin monotherapy group (n=37)			
Clinically effective	38 (41.30)	12 (32.43)	8 (36.36)	0.618	0.630
Clinically ineffective	54 (58.70)	25 (67.57)	14 (63.64)		

### 3.4 Adverse drug reactions

The incidence of ADRs, including liver dysfunction (21 cases, 22.83%), renal dysfunction (18 cases, 19.57%), pancytopenia (8 cases, 8.70%), hypokalemia (4 cases, 4.35%), and visual disturbances (2 cases, 2.17%), was lowest in the voriconazole monotherapy group. The incidence of ADRs in the caspofungin monotherapy group ranked second (67.57%), and the common ADRs were liver dysfunction (11 cases, 29.73%), renal dysfunction (9 cases, 24.32%), pancytopenia (7 cases, 18.92%) and hypokalemia (4 cases, 10.81%). The incidence of ADRs, including pancytopenia (6 patients, 27.27%), renal dysfunction (6 patients, 27.27%), liver dysfunction (4 patients, 18.18%), hypokalemia (3 patients, 13.64%), and visual disturbances (2 patients, 9.09%), was highest in the combination therapy group.

The results revealed no statistically significant differences in total ADR incidence (*P*=0.109) or the incidence of renal dysfunction (*P*=0.506), liver dysfunction (*P*=0.500), visual disturbances (*P*=0.188), or hypokalemia (*P*=0.426) between the monotherapy group and the combination therapy group. However, the incidence of pancytopenia was significantly greater in the combination therapy group than in the monotherapy group (*P*=0.013). Among the three treatment regimens, the differences in the incidence of renal dysfunction (*P*=0.673), liver dysfunction (*P*=0.564), visual disturbances (*P*=0.099), and hypokalemia (*P*=0.072) were not statistically significant; however, the incidence of total ADRs (*P*=0.010) and pancytopenia (*P*=0.004), was highest in the combination group, followed by the caspofungin group and then the voriconazole group, and the differences were statistically significant (**Table 4**).

**Table 4:** Comparison of the incidence of ADRs among antifungal treatment regimens.

ADRs/Cases (%)	Monotherapy group (n=129)		Combination therapy group (n=22)	*P* value (monotherapy vs. combination therapy)	*P* value (comparison among three regimens)
	Voriconazole monotherapy group (n=92)	Caspofungin monotherapy group (n=37)			
Renal dysfunction	18 (19.57)	9 (24.32)	6 (27.27)	0.506	0.673
Liver dysfunction	21 (22.83)	11 (29.73)	4 (18.18)	0.500	0.564
Visual disturbances	2 (2.17)	0 (0.00)	2 (9.09)	0.188	0.099
Hypokalemia	4 (4.35)	4 (10.81)	3 (13.64)	0.426	0.072
Pancytopenia	8 (8.70)	7 (18.92)	6 (27.27)	0.013	0.004
Total ADRs	43 (46.74)	25 (67.57)	17 (77.27)	0.109	0.010

### 3.5 All-cause mortality

The all-cause mortality rate in the monotherapy group was 13.18% (17 cases), which was significantly lower than that in the combination therapy group (7 cases, 37.82%, *P*=0.027). The voriconazole group had the lowest all-cause mortality rate (8 cases, 8.70%), and the caspofungin group had the second highest all-cause mortality rate (9 cases, 24.32%), all of which were lower than those in the combination group (7 cases, 31.82%). The differences among the three regimens were statistically significant (*P*=0.009) (**Table 5**).

**Table 5:** Comparison of all-cause mortality among antifungal treatment regimens.

Clinical regression/cases (%)	Monotherapy Group (n=129)		Combination Therapy group (n=22)	*P* value (monotherapy vs. combination therapy)	*P* value (comparison among three regimens)
	Voriconazole monotherapy group (n=92)	Caspofungin monotherapy group (n=37)			
Death	8 (8.70)	9 (24.32)	7 (31.82)	0.027	0.009
Survival	83 (91.30)	28 (75.68)	15 (68.18)		

The results of both univariate and multivariate regression analyses showed that there was no significant correlation between the three antifungal treatment regimens, namely voriconazole monotherapy, caspofungin monotherapy, and the combination of voriconazole plus caspofungin, and all-cause mortality. That was to say, the initial choice of combination therapy could not reduce the all-cause mortality of IPA. See **Table 6**.

**Table 6:** Univariate and multivariate analyses of all-cause mortality in patients with IPA.

Variable	Univariate analysis				Multivariate analysis	
	Death (n=24)	Survival (n=127)	P-value	OR (95% CI)	P-value	OR (95% CI)
Age (year, $\bar{\text{x}}$ ± s)	72.58 ± 13.52	67.49 ± 13.31	0.092	1.032 (0.995-1.070)	–	–
Female (case, %)	12 (50.00)	40 (31.50)	0.085	0.460 (0.190-1.112)	–	–
BMI (kg/m^2^, $\bar{\text{x}}$ ± s)	20.92 ± 5.81	21.43 ± 6.27	0.074	0.649 (0.447-1.218)	–	–
History of steroid use (case, %)	8 (33.33)	73 (57.48)	0.034	2.704 (1.079-6.776)	0.026	3.043 (1.142-8.111)
Underlting conditions (case, %)					–	–
Hypertension	9 (37.50)	46 (36.22)	0.905	0.947 (0.384-2.333)	–	–
Coronary heart disease	7 (29.17)	42 (33.07)	0.708	1.200 (0.462-3.117)	–	–
Type 2 diabetes	12 (50.00)	61 (48.03)	0.860	0.924 (0.386-2.212)	–	–
Liver failure	5 (20.83)	12 (9.45)	0.115	0.397 (0.125-1.253)	–	–
Renal failure	6 (25.00)	5 (3.94)	0.001	8.130 (2.247-29.412)	0.001	9.434 (2.421-37.037)
COPD	13 (54.17)	61 (48.03)	0.582	0.782 (0.326-1.876)	–	–
Bronchiectasis	4 (16.67)	14 (11.02)	0.437	0.619 (0.185-2.075)	–	–
Charlson comorbidity index ( $\bar{\text{x}}$ ± s)	4.89 ± 3.12	4.24 ± 2.78	0.087	1.048 (0.902-1.579)	–	–
Antifungal regimen			0.890	0.951 (0.469-1.928)	0.792	0.905(0.431-1.899)
Voriconazole	8 (33.33)	84 (66.14)			–	–
Caspofungin	9 (37.50)	28 (22.05)			–	–
Combination therapy	7 (29.17)	15 (11.81)			–	–
Treatment duration (day, $\bar{\text{x}}$ ± s)	7.04 ± 4.17	7.85 ± 5.52	0.495	0.967 (0.878-1.065)	–	–
APACHE II (score, $\bar{\text{x}}$ ± s)	18.17 ± 9.29	16.12 ± 5.84	0.091	1.112 (0.847-1.756)	–	–

## 4 Discussion

IPA is the most common type of IFD in patients in the ICU and is characterized by atypical clinical symptoms, such as cough, sputum production, shortness of breath, chest tightness, and fever, which are often indistinguishable from the symptoms of other pulmonary infections, leading to frequent misdiagnosis or delayed diagnosis (Lei et al., 2020). Although IPA is traditionally associated with immunocompromised patients, recent studies have shown that immunocompetent patients are also at risk, with an increasing incidence in such patients observed in recent years, requiring the attention of clinicians and pharmacists (Wang, 2022).

IPA progresses rapidly and has a high mortality rate. Studies report that, without aggressive treatment, it can lead to patient death within 7–14 days (Lei et al., 2020). Therefore, timely diagnosis and prompt antifungal treatment are crucial for improving outcomes in IPA patients (Tian et al., 2020). Voriconazole is a triazole antifungal agent that works by inhibiting the synthesis of ergosterol, a necessary component of the fungal cell membrane, thus disrupting the integrity of the fungal cell and achieving antifungal effects (Zhang and Ma, 2019; Li et al., 2021). It is the first-line treatment for IPA recommended by the IDSA. Caspofungin is an echinocandin antifungal agent that inhibits the synthesis of β-(1,3)-D-glucan in the cell walls of *Aspergillus* species, making it an alternative or salvage treatment for IPA, as recommended in the guidelines (Hou and Xu, 2017). In this study, a majority of the 151 IPA patients initially received voriconazole monotherapy, in accordance with the guidelines. Additionally, 25% of patients were treated with caspofungin monotherapy due to good initial empirical treatment response, good liver function at admission, or physician habits and experience. The combination of voriconazole and caspofungin was less frequently used.

When treating IPA, obtaining accurate pathogen information or understanding the epidemiological characteristics of local *Aspergillus* strains is of utmost importance (Herrera et al., 2025), as different *Aspergillus* species exhibit varying sensitivities to drugs, and some even have innate resistance. For instance, most *Aspergillus* species are sensitive to amphotericin B, while *A. terreus* has innate resistance to amphotericin B, and *A. flavus* can develop acquired resistance (Su et al., 2019). MALDI-TOF MS is a novel soft ionization mass spectrometry technique for the identification of clinical microbial pathogens. It detects the protein fingerprint of microorganisms and compares the unique mass spectral peaks of different fungal species with the mass spectrometry database to quickly obtain microbial identification results. It demonstrates advantages in fungal homology analysis, pathogen typing, virulence factor identification, and drug resistance detection. The research findings of Zhang et al. showed that the accuracy rate of MALDI-TOF MS in identifying filamentous fungi to the species level was 92.9%, which was significantly higher than that of traditional manual identification methods. In our study, MALDI-TOF MS was used to identify *Aspergillus* species. The results revealed that in this region, *A. fumigatus* was the most common pathogenic fungus, accounting for approximately 70% of the fungal community. This was followed by *A. flavus, A. niger, A. terreus* and *A. nidulans.* This finding was essentially consistent with the reported epidemiology of *Aspergillus* species in mainland China (Khan et al., 2024). Previously, epidemiological studies on *Aspergillus* species have been conducted in China and other countries (Chen et al., 2018; Ma et al., 2019; Romero et al., 2019). In mainland China, *A. fumigatus* has always been identified as the most common *Aspergillus* species, accounting for 75.14% (Wang et al., 2019). Among the non-*A. fumigatus* species, *A. flavus* (12.25%) has the second highest prevalence rate, followed by *A. niger* (6.14%), *A. terreus* (0.82%) and *A. nidulans* (3.96%). *A. flavus*, the second most common non-*A. fumigatus* pathogen, had been reported in many countries (Lestrade et al., 2019; Pfaller et al., 2022). In contrast, in some countries, *A. niger* and *A. terreus were* considered the second most common *Aspergillus* species (He et al., 2012). Aspergillosis is mainly caused by *A. fumigatus.* However, some reported cases in China had shown that aspergillosis can also be caused by non-*A. fumigatus* species (Shen et al., 2022). This indicateed that *A. fumigatus* was not the sole pathogen of aspergillosis.

Voriconazole is the first-line treatment for invasive aspergillosis (IA) and has become the main treatment method in the past 20 years. Studies have reported that it can significantly improve the treatment outcome of patients at 12 weeks (Herbrecht et al., 2002; Ullmann et al., 2018). Caspofungin is an alternative treatment for IA, but it has never been evaluated as a monotherapy for IA in a randomized controlled trial (RCT) (Herrera et al., 2025). It is usually recommended that after the initial treatment regimen fails, other types of drugs should be selected for monotherapy or combination therapy (Patterson et al., 2016; Tissot et al., 2017). In this study, the efficacy of the initial choice of monotherapy or combination therapy was comparable. Further research revealed that the clinical effectiveness rate of voriconazole monotherapy was essentially comparable to that of caspofungin monotherapy and the combination of voriconazole plus caspofungin. That is, in our study, the clinical efficacy of these three regimens for IPA was essentially the same. This finding is consistent with the results of a study published by Yang et al. in 2021, which included 35 critically ill patients with IPA. They reported that, compared with voriconazole monotherapy, the initial choice of the combination of voriconazole plus caspofungin did not improve the clinical treatment effect in patients (Yang et al., 2021). A systematic review involving 1,071 patients revealed that for the treatment of primary IPA, the cumulative evidence regarding the use of combination antifungal therapy is contradictory. Among them, two studies demonstrated that the outcome of combination antifungal therapy was better than that of monotherapy, one study showed a trend toward a better outcome, and the remaining two studies indicated that combination antifungal therapy had no additional advantage over monotherapy or that the response to monotherapy was better (Garbati et al., 2012). Another meta-analysis including 16 studies and 1,833 patients revealed that the combination of echinocandins with triazoles or amphotericin B, compared with nonechinocandin monotherapy, improved the clinical outcomes of salvage treatment (Panackal et al., 2014). The results of a study published by Yang et al. in 2023, which included 60 patients with neutropenia complicated by IFD among patients with malignant hematological diseases, showed that the combination of caspofungin with voriconazole or amphotericin B was acceptable in terms of effectiveness and safety (Yang et al., 2013). The results of a meta-analysis published in 2015 on the safety and effectiveness of caspofungin for the treatment of IFD revealed that, for confirmed IPA, the therapeutic effect of caspofungin monotherapy was comparable to that of voriconazole monotherapy (Li, 2015), which is consistent with the results of this study. In conclusion, multiple studies have reported contradictory findings regarding the choice of treatment regimens between monotherapy and combination therapy. This is considered related to differences among patient populations, the lack of specific clinical characteristics, and differences in underlying diseases. However, for the initial treatment of IPA, a monotherapy regimen of either voriconazole or caspofungin is still recommended. Once the initial treatment fails, a combination therapy regimen of voriconazole combined with caspofungin can be considered.

An analysis of ADRs associated with various antifungal drugs revealed that the ADRs associated with voriconazole mainly manifest as visual abnormalities, rashes, abnormalities in liver and kidney functions, and pancytopenia (Tang and Liu, 2020). In addition, the prolongation of the QT interval (QTc) inherent in the triazole family in general and drug–drug interactions may also be clinical issues that require attention (Maertens et al., 2021). The ADRs associated with caspofungin mainly involve the systemic system, including elevated transaminase levels, chills, nausea, vomiting, fever, rashes, and pancytopenia (Liu et al., 2018). The results of our study revealed that monotherapy, including voriconazole monotherapy or caspofungin monotherapy, was comparable to the combination therapy with voriconazole and caspofungin in terms of the incidence of total ADRs, liver and kidney function impairment, visual abnormalities, and electrolyte metabolism abnormalities. However, the combination therapy significantly increased the incidence of pancytopenia. Therefore, clinicians and pharmacists should strengthen the monitoring of the routine blood test results of critically ill patients with IPA who are receiving the combination therapy.

In terms of all-cause mortality, the combination therapy group had a significantly higher mortality rate than did the monotherapy groups. However, this result should not be interpreted as indicating that combination therapy is a predictor of all-cause mortality. Rather, it is likely related to the severity of the underlying disease and the progression of the condition, as patients who received combination therapy generally presented with more severe symptoms, as indicated by their higher APACHE II scores. This suggests that the initial choice of combination therapy typically reflects more severe disease, thus leading to a higher mortality rate. Nonetheless, this study also confirmed that the initial combination therapy did not improve patient prognosis or reduce all-cause mortality, which was consistent with the results of the RCT conducted by Kieren et al (Marr et al., 2015). They included 454 patients with suspected or confirmed IA infection and compared the efficacy of the combination of echinocandins plus voriconazole with that of voriconazole monotherapy. The results showed that in terms of reducing the 6-week mortality rate of patients, the effectiveness was limited, and no statistically significant results were achieved; thus, a clear conclusion of superiority could not be drawn.

This study has certain limitations. First, as this was a retrospective study, there were differences in data homogeneity, and some data were missing, which could have introduced bias into the results. Second, the study data were collected from two centers, meaning that the results could be influenced by the level of diagnostic and therapeutic expertise at the two institutions, which may have limited the generalizability of the findings. Additionally, alternative therapies recommended in the guidelines, such as amphotericin B and itraconazole, were infrequently used in this study, and given potential data bias, they were not included in the analysis, making it impossible to clarify their efficacy and safety. Finally, the relatively small number of patients included in this study may have impacted the robustness of the results. We will continue to supplement and analyze these data in the future.

In conclusion, in critically ill patients with IPA in the ICU, both voriconazole monotherapy and caspofungin monotherapy were found to be clinically effective and safe. The combination of voriconazole and caspofungin did not offer additional benefits in terms of clinical efficacy or overall survival. Moreover, combination therapy significantly increased the incidence of pancytopenia and did not improve all-cause mortality or prognosis. Therefore, voriconazole monotherapy remains the first-line treatment for IPA in critically ill patients because of its clinical efficacy and safety profile. Caspofungin monotherapy is a viable alternative with comparable efficacy and safety. The combination of voriconazole and caspofungin is not recommended as an initial treatment regimen for IPA in critically ill patients. Further randomized, controlled, multicenter, large-scale clinical trials are needed to confirm the efficacy and safety of various antifungal regimens in critically ill IPA patients.

## Data Availability

The original contributions presented in the study are included in the article/supplementary material. Further inquiries can be directed to the corresponding author.
